# Local Ecological Knowledge and Cultural Perceptions of Snakes in Sudan

**DOI:** 10.1002/ece3.72959

**Published:** 2026-01-18

**Authors:** Rania M. H. Baleela, Muhammad E. M. O. Elamin, Abubakr Mohammad, Sara A. K. Saeed

**Affiliations:** ^1^ Toxic Organisms Research Centre, Faculty of Science University of Khartoum Khartoum Sudan; ^2^ Department of Zoology, Faculty of Science University of Khartoum Khartoum Sudan; ^3^ National Poisons Information Service (Birmingham Unit) Birmingham UK; ^4^ Conflict and Environment Observatory West Yorkshire UK; ^5^ Sudan Natural History Museum, Faculty of Science University of Khartoum Khartoum Sudan

**Keywords:** cultural beliefs, human—wildlife interactions, Local Ecological Knowledge, natural heritage, snake conservation, Sudan

## Abstract

Local Ecological Knowledge (LEK) is central to biodiversity conservation and public health, particularly in regions where human–snake interactions are frequent. In Sudan, snakes hold complex cultural meanings: valued as rodent predators, feared for venom, and linked to spiritual beliefs. To document LEK and attitudes toward snakes, we conducted a validated Arabic language online survey (*n* = 192) across 16 states (Feb–Apr 2025). Results indicated that encounters with snakes were most common during the rainy season (*n* = 129, 53%), especially in agricultural areas (*n* = 72, 38%), with the genus *Naja* (33%) and *Echis* (25%) most frequently reported. Identification of snakes by community members relied on coloration, and 32% of respondents could not distinguish venomous from nonvenomous species. Conservation attitudes regarding snakes were polarized, with similar proportions strongly supportive of (31%) and 29% opposed to snake conservation; yet 95% endorsed integrating LEK with science to promote coexistence. Despite this, 65% of respondents reported killing snakes regardless of venom status. Beliefs commonly described snakes as *jinn* (i.e., demons) (47%) or avengers (25%). Reported treatments included incision and suction (31%), use of diverse treatments (29%), fang extraction was reported by 14% of respondents, and in nearly all of these cases the “fangs” was extracted using plant poultices, except for one report where frogs were used instead. 5% reported use of a venom stone, no knowledge of treatments (19%) and rare access to antivenom. In conclusion, perceptions of snakes are shaped by a complex interplay of ecological observations, cultural beliefs, and practical health responses. Conservation and public health strategies should integrate LEK with scientific knowledge, strengthen evidence‐based snakebite management, and tailor conservation and health interventions to regional contexts.

## Graphical abstract

In Sudan, snakes are viewed through a mix of ecological, cultural, and spiritual lenses, shaping both conservation attitudes and health responses. Our survey (*n* = 192) across 16 states revealed frequent encounters, polarized conservation views, widespread killing of snakes, reliance on traditional remedies, and strong public support (95%) for integrating local and scientific knowledge to improve coexistence and snakebite management.

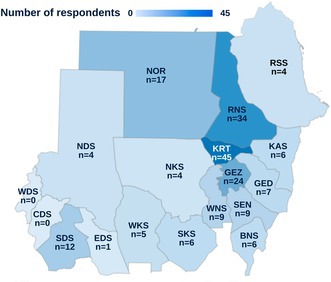

## 1 Introduction

Local Ecological Knowledge (LEK) encompasses the knowledge, practices, and beliefs acquired through extensive personal observation and interaction with local ecosystems. It is shared among local resource users, often within indigenous communities, and is typically transmitted across generations (Charnley et al. 2008; Grimm et al. 2024). To achieve a comprehensive understanding of ecology and evolution, the invaluable contributions of LEK must be recognized and embraced (Akani et al. 2013; Reid et al. 2024; McKinnon and Muth 2024). These knowledge systems, rooted in deep connections to land and culture, provide unique ecological insights that can only be fully realized through respectful collaboration with indigenous communities (Reid et al. 2024). Globally, indigenous peoples have sustained biodiversity and managed natural resources with profound wisdom for millennia (Garnett et al. 2018), highlighting the urgent importance of integrating these knowledge systems into contemporary conservation practice (Ogar et al. 2020).

In Sudan, snakes hold a complex position within both the natural world and cultural beliefs, requiring careful consideration for conservation efforts. They are recognized for their ecological role, particularly as predators of rodents, yet they are also feared due to the risk of venomous bites, especially among farm workers. Snakes are also respected in relation to ancient religious and spiritual beliefs. Snakes feature prominently in spiritual practices in various regions of Sudan. In Jebel Daier, South Kordofan, they are revered in multiple spiritual forms (Bolton 1936; Kafi 2006). Ancient snake rituals are evident in Jebel Sileia, as indicated by snake rock engravings discovered in the 1950s (Paul 1954), and at the pinnacle and temples of Jebel Barkal in the north (Kendall and Mohamed 2022). In pre‐Islamic Taqali, snakes were worshipped and even offered human sacrifices (Dawru 1994; Muhyiddin 2021). Other indigenous traditions, particularly those associated with slavery and enslaved peoples from the south and Nuba Mountains (Kenyon 1991, 2012; Makris 1994), link snakes with spiritual practices and *Zar* rituals, associating them with healing and protection (Makris 1994), possibly reflecting an early form of snake, python, or Egyptian cobra worship. In contrast, in post‐Christian and Islamic traditions, Sudanese communities increasingly associated snakes with sin and demonic forces.

Sudan is rich in snake fauna, with 70 species recorded or suspected to be present, reflecting the country's diverse habitats and ecological regions (Spawls and Branch 2020; Spawls et al. 2023; Abd El‐Rahman et al. 2023; Baleela et al. 2024; Baleela et al. 2025; Toxic Organisms Research Centre (TORC), Unpublished). This rich diversity is accompanied by extensive traditional and local knowledge about snake ecology and snakebite treatment. However, this knowledge remains largely undocumented and is at risk of being lost.

The understanding of cultural and community perceptions of snakes in Sudan is vital for conservation efforts. It will help inform and guide future work which may either embrace community values or respectfully work toward introducing positive changes. For conservation strategies to succeed, they should address both the ecological importance of snakes and the surrounding cultural context. This can be achieved by bridging scientific herpetological data with community‐held knowledge. For example, research from southern Nepal shows that fear, misconceptions, and limited understanding of snake ecology strongly influence people's behavior, leading to unnecessary killing of snakes and increased snakebite risk. By pairing ecological information with targeted community education that responds to these beliefs and knowledge gaps, conservation efforts can both protect snake populations and improve human safety (Pandey et al. 2016). The same was observed in Jordan (Eid et al. 2021).

This study aims to document local/cultural attitudes toward snakes to inform conservation of Sudan's biocultural heritage while also improving snakebite management and conservation outcomes in communities. It highlights how citizen knowledge and integration of LEK can inform both ecological understanding and public health initiatives in biodiverse regions.

Our study aimed to address the gap in documentation of LEK by surveying Sudanese citizens to document: (a) local ecological knowledge about snake distribution and behavior; (b) current perceptions and beliefs regarding snakes; (c) traditional practices for snakebite treatment and prevention; (d) community awareness and educational needs concerning human–snake interactions; (e) approaches to snakebite treatment and management.

## 2 Materials and Methods

### 2.1 Study Design

An online, Google Forms survey, in Arabic, was designed to collect data on Sudanese respondents' LEK about snakes, using a modified snowball sampling method (Ting et al. 2025). The questionnaire was validated through a pilot test involving 10 Sudanese volunteers from different backgrounds to assess its content validity, applicability, suitability, and consistency. The volunteers completed the questionnaire and provided feedback that helped refine its structure and wording. The final questionnaire comprised 27 sections, including items tailored to specific locations and thematic sets of questions designed to assess participants' knowledge about snakes and their attitudes toward them. Both closed‐ and open‐ended formats in addition to one Likert scale question were used to capture quantitative and qualitative data that covered background demographic data, snake and snakebite related knowledge, traditional treatments and related cultural aspects and perceptions. Informed consent was obtained from each participant before participation.

The survey was structured as follows: (S1: Translated questionnaire in English):

*Section 1* introduced the context of the study and explained what participants were consenting to share.

See Supporting Information for the full questionnaire.

*Sections 2–20*: focused on state‐dependent questions on geographic location for which the participant was providing knowledge about snakes.

*Section 21*: asked about snake ecology, encounters, and traditional beliefs and practices. Respondents were provided with both close‐ended and open‐ended questions to capture detailed information.

*Sections 22–26*: gathered information on traditional medicine and practices used to treat snakebite envenomation. Respondents were provided with both close‐ended and open‐ended questions to capture detailed information. For example, in the questions on traditional treatments, respondents could select from a list of commonly used plants and report additional plants not included in the list. All plant names were provided in Arabic, and based on our knowledge of these local names, the corresponding scientific names were accurately assigned and are reported in the manuscript.

*Section 27*: addressed participants' awareness and education concerning snakes and snakebite management.

### 2.2 Study Sample and Participants

The questionnaire targeted residents of 18 Sudanese states. It was distributed through multiple social media and electronic channels to maximize reach. It was also shared via the Toxic Organisms Research Centre Facebook group (TORC 2023–2025) and circulated among colleagues, who were encouraged to share it with their acquaintances. Facebook group members were also encouraged to further disseminate the questionnaire. Additionally, the survey was sent to large professional WhatsApp and Telegram groups by team members, with participants encouraged to share it within their communities. Participants from cultural clubs were asked to recruit their members, and local NGO staff assisted in recruiting respondents in remote or conflict‐affected areas, such as the Darfur states.

Participants in the study were requested to respond transparently and anonymously, unless they chose to provide their contact details for potential participation in future studies. The survey was launched on 6th February 2025, and data collection continued until 2nd April 2025, following which no further responses were received.

### 2.3 Statistical Analysis

Quantitative data were analyzed using Microsoft Office Excel, beginning with an overall assessment of the dataset, followed by separate analyses for each question category to summarize demographic distributions and general response patterns. Qualitative data were analyzed using thematic analysis, where raw responses were read thoroughly, main themes identified and then statements were manually coded into main themes in Microsoft Office Excel (Example in Table 1). Zoho Analytics (Zoho 2025) was used to visually explore the geographic distribution of encoded themes, after which theme frequencies per state were computed in WPS Office Sheets and visualized using bar graphs generated in the same software.

**TABLE 1:** Thematic coding of beliefs about snakes: an example of the coding process.

Statement	Thematic code
The family considers it a bad omen to mention the snake, and they believe that the father takes revenge on the killer	Bad omen+ Revenge
Don't mention it at night	Jin
Ab Lamba, who misleads travelers at night	Jin
The snake records the image of its killer in its eyes	Killer
It is claimed that the black‐colored snake is from the jinn, and it should not be killed	Jin
Large snakes are believed to be a type of jinn	Jin
Some snakes bite on Fridays, some break the nasal passages like the ‘Aslah’ (a specific type), and some wait until a person goes into the water and then coil around their legs	Day connections
The snake records the image of its killer in its eyes	Killer
It could be from the jinn	Jin
An attempt to provoke certain long‐necked birds by saying that the snake has a longer neck than theirs, causing the birds to walk and raise their necks higher, making them longer. This is done with a rhythmic beat	Association with birds

For inferential statistical analysis, a chi‐square test of independence was conducted using Stangroom's online chi‐square calculator (Stangroom 2025). Prior to analysis, the statistical assumptions of the chi‐square test were verified: all variables were categorical, observations were independent, and no expected cell frequency fell below 5; therefore, the test's assumptions were met. The calculator was used exclusively for the computation of the chi‐square statistic, *p*‐value, expected counts, and standardized residuals; data tabulation and preparation were performed in Excel and WPS Office Sheets. Statistical significance was set at *α* ≤ 0.05.

## 3 Results

**FIGURE 1:**
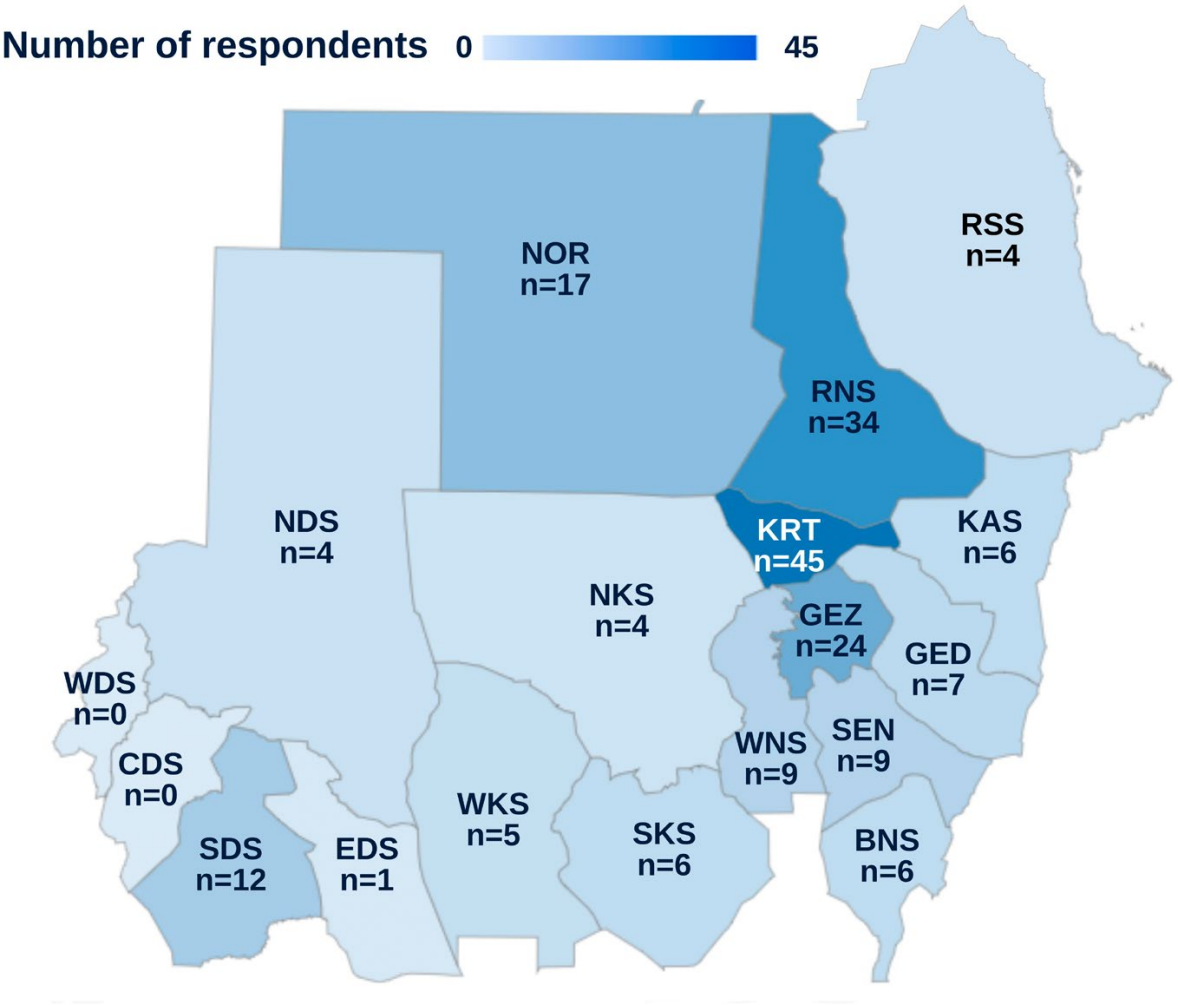
Geographic distribution of survey respondents across Sudanese states. The map shows the number of participants from each state, with darker shades of blue representing higher response rates. The highest number of respondents was recorded in Khartoum (KRT) and River Nile (RNS) states, while fewer responses were received from western and southern states. Key to states: BNS = Blue Nile state, CDS = Central Darfur state, EDS = East Darfur state, GED = Gedaref state, GEZ = Al Jazirah state, KAS = Kassala state, KRT = Khartoum state, NDS = North Darfur state, NKS = North Kordofan state, NOR = Northern state, RNS = River Nile state, RSS = Red Sea state, SDS = South Darfur state, SEN = Sennar state, SKS = South Kordofan state, WDS = West Darfur state, WKS = West Kordofan state, WNS = White Nile state.

**FIGURE 2:**
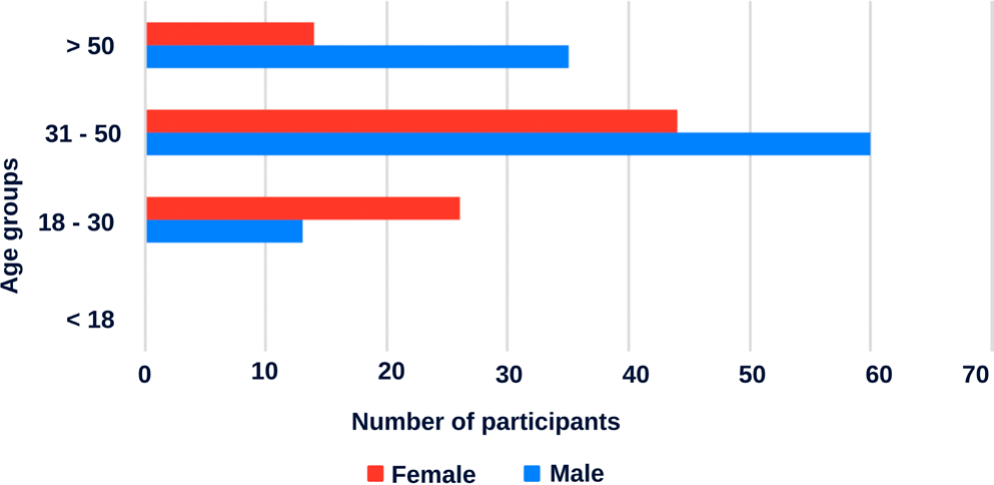
Demographic distribution of respondents by age group and gender. The horizontal bar chart shows the number of male (blue) and female (red) participants across four age categories. Most respondents were aged 31–50 years, followed by those aged 18–30 years, with relatively fewer participants aged over 50 and none under 18 years.

A total of 192 participants, representing 16 of the 18 Sudanese states, answered the online survey (Figure 1). The majority of the respondents (*n* = 104; 54%) fell in the age group 31–50 years (Figure 2).

### 3.1 Snake Ecology, Encounters, Identification, Attitudes Toward Snakes, and Conservation

**FIGURE 3:**
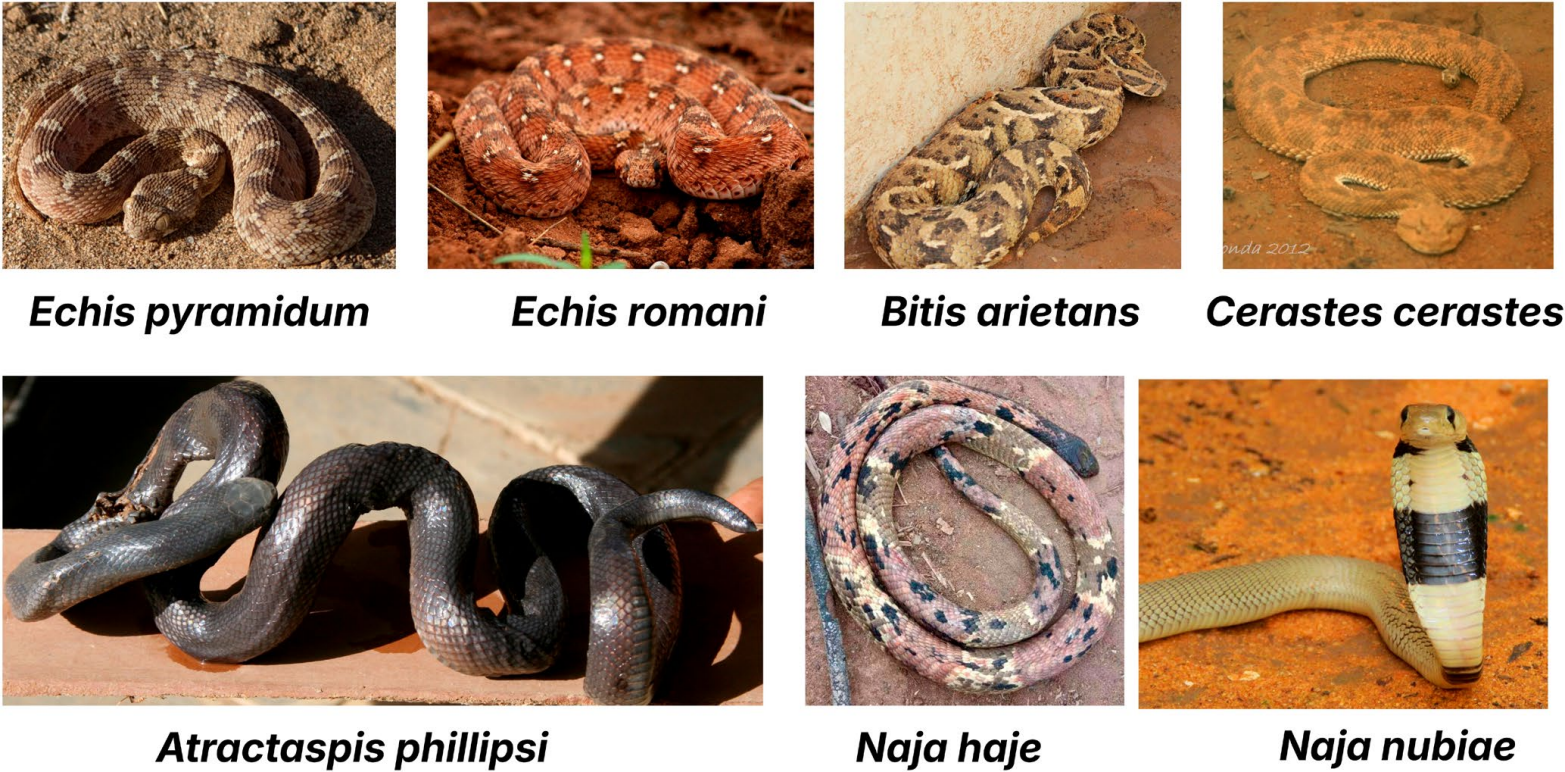
Representative venomous snake species of Sudan. Top row (left to right): *Echis pyramidum, Echis romani, Bitis arietans, Cerastes cerastes*. Bottom row (left to right): *Atractaspis phillipsi, Naja haje, Naja nubiae*. All species are medically relevant and found in varied habitats across the country. Photos credit: *Echis pyramidum, Echis romani, Cerastes cerastes, Atractaspis phillipsi, Naja haje*: @Abubakr Mohamed. *Naja nubiae* @Mohamed Salah and *Bitis arietans* @Sudan Natural History Museum.

**FIGURE 4:**
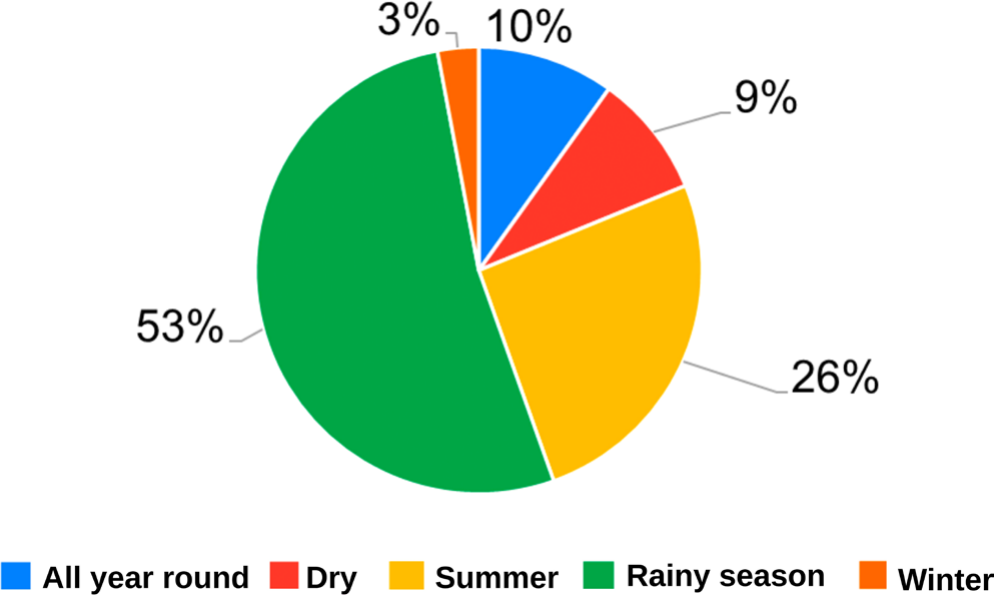
Seasonal variation in snake encounters: Respondents reported peak snake encounters predominantly during the rainy season (53%), followed by summer (26%), consistent activity throughout the year (10%), the dry season (9%), and winter (3%), reflecting periods of reduced activity due to brumation or dormancy.

Sudan is home to 70 snake species, of which 15 are of high medical importance. The most frequently encountered venomous snake species in Sudan are listed in Table 2, Figure 3. Snake encounters were reported most frequently during the rainy season months of June–September (*n* = 129, 53%), followed by the summer months of March–June (*n* = 63, 26%), with some participants noting their presence throughout the year (*n* = 24, 10%) (Figure 4). Ninety‐nine participants (52%) indicated that snakes are abundant in their area.

**TABLE 2:** Most frequently encountered venomous snake species in Sudan as observed by TORC (unpublished data).

	Family	Species	Common name
1	Atractaspididae	*Atractaspis phillipsi* Barbour, 1913	Phillips' Burrowing Asp, Sudan burrowing asp
2	Viperidae	*Echis pyramidum* (Geoffroy Saint‐Hilaire, 1827)^a^	North‐East African Carpet Viper
3	Elapidae	*Naja haje* (Linnaeus, 1758)	Egyptian cobra
4	Elapidae	*Naja nubiae* (Wüster & Broadley, 2003)	Nubian spitting cobra
5	Viperidae	*Bitis arietans* (Merrem, 1820)	Puff adder
6	Viperidae	*Cerastes cerastes* (Linnaeus, 1758)	Desert Horned Viper

^a^ Most snakebite fatalities in the country are attributed to this species (Baleela et al. 2025).

The most areas where snakes were encountered by survey participants were agricultural areas (*n* = 72, 38%), followed by open uninhabited areas (outside villages) (*n* = 33, 17%) (Table 3).

**TABLE 3:** Reported snake habitats, species, and seasonal patterns by state in Sudan.

State	Gender		Areas preferred by snakes (mentions^a^)							Species mentioned									Season				
	Male	Female	Agricultural areas	Near water	Khalaa	Near houses	Forests	Sand dunes	Other^b^	Echis	Naja	Atractaspis	Psammophis	Bitis	Cerastes	Python	Burrowing snake	Causes	Rainy	Summer	Dry	All year round	Winter
GEZ	11	13	19	6	14	9	7	3	1	2	3	1	2	0	0	0	0	0	13	11	6	3	1
GED	6	1	6	2	4	0	2	1	1	3	2	0	1	0	0	0	0	0	5	2	1	1	0
BNS	3	3	5	1	5	2	2	1	1	0	2	0	0	0	0	0	0	0	5	0	1	0	0
EDS	1	0	1	0	1	0	1	1	0	1	0	0	0	0	0	0	0	0	1	0	0	0	0
KAS	3	3	6	5	3	4	2	1	0	1	4	1	0	1	0	0	0	0	3	2	1	2	0
KRT	26	19	32	11	7	6	12	1	3	2	2	1	0	0	0	0	1	0	37	20	3	1	0
NDS	2	2	2	1	2	0	1	2	1	0	0	0	0	0	0	1	0	0	1	2	0	0	1
NKS	3	0	2	1	1	0	2	1	0	1	2	1	0	0	0	0	0	0	3	2	0	0	0
NOR	6	11	13	4	5	8	2	5	0	0	4	0	1	0	3	3	0	0	6	8	1	6	0
RSS	4	0	2	1	2	0	2	1	0	1	0	0	1	0	0	0	1	0	3	1	1	0	1
RNS	12	22	27	7	8	8	8	6	1	0	6	0	0	0	0	2	4	0	19	14	5	9	2
SEN	6	3	6	3	4	1	3	0	0	6	4	3	1	2	0	1	0	1	8	1	0	0	0
SDS	11	1	8	1	1	0	6	3	0	8	3	1	4	0	0	1	0	0	9	2	1	0	1
SKS	5	1	5	3	1	2	5	1	3	3	5	1	1	3	0	2	0	0	5	1	0	0	1
WKS	4	1	2	0	4	0	2	0	0	4	1	0	0	1	0	0	0	0	4	1	1	1	0
WNS	5	4	7	2	4	0	1	0	2	1	2	0	0	0	0	0	2	0	7	1	0	1	0
Total	108	84	143	48	66	40	58	27	13	33	40	9	11	7	3	10	8	1	129	68	21	24	7

Abbreviations: BNS = Blue Nile state, DAR = Darfur states, GED = Gedaref state, GEZ = Al Jazirah state, KAS = Kassala state, KRT = Khartoum state, KOR = Kordofan states, NOR = Northern state, RSS = Red Sea state, RNS = River Nile state, SEN = Sennar state, WNS = White Nile state. The largest observed values are highlighted in grey.

^a^ Mentions = number of mentions. Some participants choose more than one area.

^b^ Other locations included: trees, under barrels, inside houses and storing rooms, in the mountain areas and under rocks.

Based on personal experiences and familiarity with local species, 80 (41.7%) of the 192 participants indicated that they knew about the presence of highly venomous snakes in their areas. Seventy‐nine of this cohort (98.75%) listed the local name of eight species (at least one species per participant summing up to 123 mentions). *Naja* spp. had the highest number of mentions within the 123 mentions (*n* = 41, 33% of mentions), followed by *Echis* spp. (*n* = 31, 25% of mentions) (Table 3), followed by *Psammophis* and *Python* spp. (*n* = 11 each, 9% of mentions), followed by *Atractaspis* and burrowing snakes spp. (*n* = 8 each, 7% of mentions). The least mentioned highly venomous snake was the horned desert viper *Cerastes cerastes* (*n* = 3, 2%).

Attitudes toward snake conservation were mixed: a highly polarized result was obtained for the participants’ opinion regarding the following statement: ‘Snakes are important creatures that should be protected.’ A near balance between strong support (*n* = 59, 31%) and strong opposition (*n* = 56, 29%) was obtained (*χ* ^2^‐test value = 42.219, *p*‐value < 0.00001); only 18 (9.4%) of the participants agreed with the statement and 17 (8.9%) disagreed, whereas 42 (22%) were neutral. Despite these attitudes, 95% (*n* = 183) believed that integrating traditional and scientific knowledge could improve snake protection and create a better human‐snake coexistence.

In terms of identification, 53 (28%) of respondents relied on color or patterns; 14% identified snakes according to the color or patterns or the size or shape of the head (*n* = 27), 17% based on the head shape or size (*n* = 32), while 32% (*n* = 80) reported not knowing how to distinguish venomous from nonvenomous snakes or answered “I don't know”.

Of the respondents, 43% (*n* = 82) reported traditional methods for snake identification, most commonly from River Nile state (*n* = 19, 23%), while no such practices were reported in North Darfur, White Nile, and Red Sea states. Khartoum contributed the highest number reporting absence of traditional identification methods (*n* = 45, 23%). Community attitudes showed that 65% (*n* = 124) kill snakes regardless of venom status, 29% (*n* = 56) avoid them, 3.6% (*n* = 9) hunt them, 1.6% (*n* = 4) coexist with them, and one respondent (~1%) indicated respect or worship of snakes. Only 30% (*n* = 58) noted the presence of local snake‐handling experts in their communities. The vast majority (*n* = 164, 85%) had never received training or awareness on snakes or snakebite management, with Khartoum (*n* = 42, 22%) and River Nile (*n* = 27, 14%) contributing the largest shares. Strikingly, just 4% (*n* = 7) of participants were aware of any active scientific or government programs addressing snakes in their areas.

**FIGURE 5:**
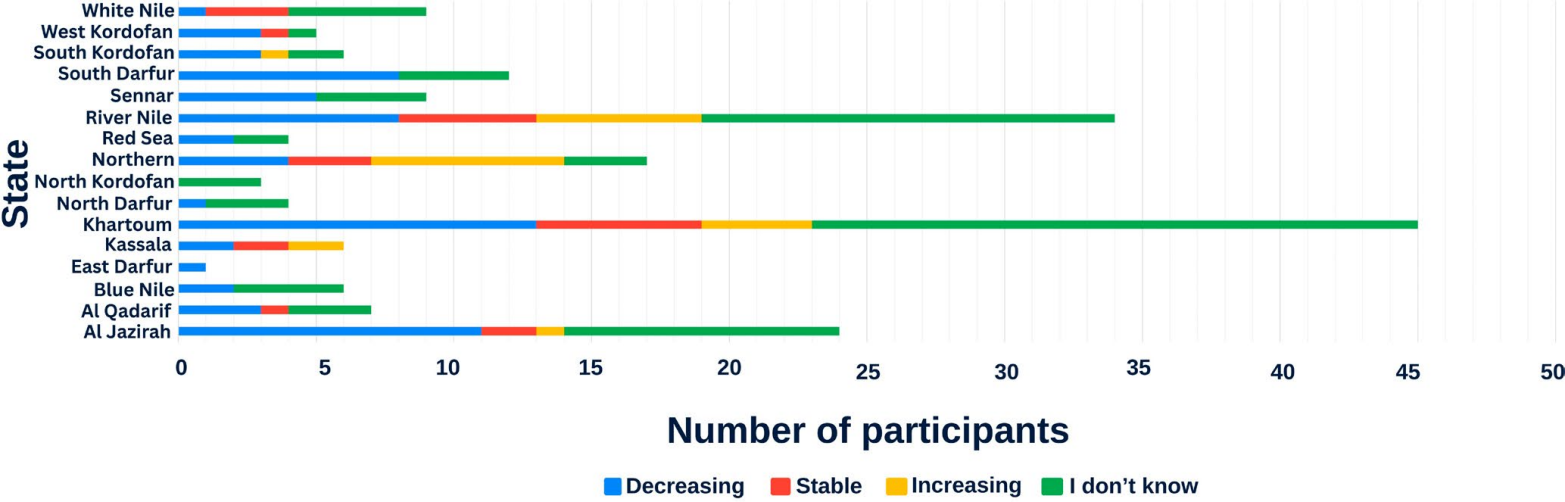
Trends in snake numbers over time across Sudanese states. Bars represent the proportion of respondents reporting a decrease, stability, or increase in the number of snakes encountered, as well as those responding “I don't know.” Responses vary markedly by state, with the highest levels of uncertainty reported in Khartoum and River Nile states.

Most respondents were uncertain about trends in snake populations over time (42%, *n* = 81). Among the remainder, 35% (*n* = 67) perceived a decline in population, 12% (*n* = 23) reported stability, and 11% (*n* = 21) perceived an increase (Figure 5). When asked specifically about the impact of the ongoing war on snakes' numbers or behavior, 72% (*n* = 138) reported no knowledge, while 12% (*n* = 23) observed changes—primarily from River Nile (30%) and Khartoum (26%) states. Regarding snake diet, rodents were most frequently cited as prey (33%, *n* = 64), followed by birds (17%, *n* = 37).

### 3.2 Traditional Beliefs and Practices

**FIGURE 6:**
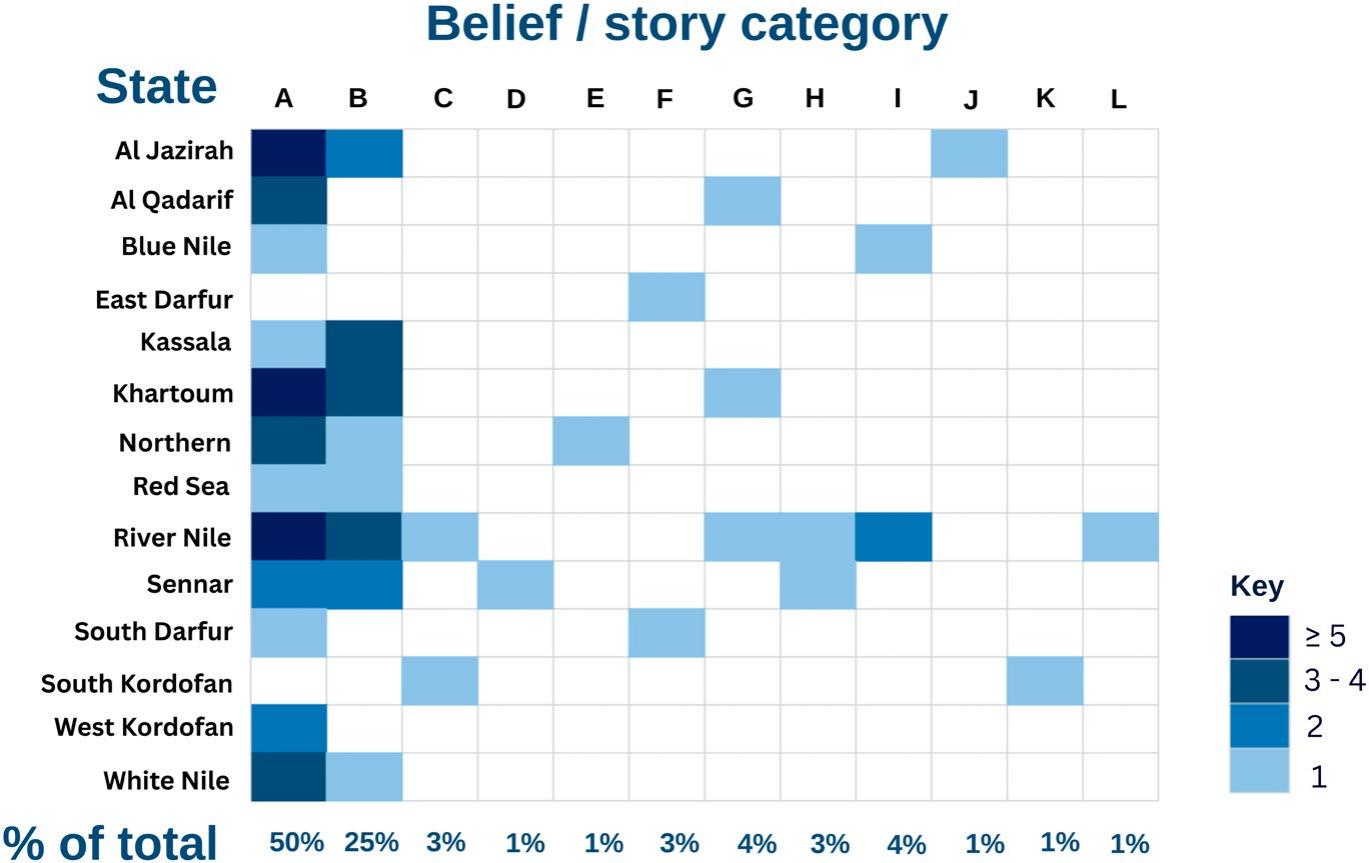
Heatmap showing the reported beliefs and stories (categories A–L) across Sudanese states. Darker shades indicate higher numbers of reports, while the bottom row shows each category's share (%) of the total reports. (A) Jinn/demons; (B) avengers; (C) Bad omen/bad karma; (D) Good omen of rain and prosperity; (E) Ancestor; (F) daily connection of bites, where participants' perceptions of snakebite timing show the belief that bites occur only on specific days, such as Wednesdays or Fridays; (G) guardians; (H) have a precious jewel in their heads to attract insects; (I) connected with birds' behavior; (J) beseech the holy Sheikh Alkabbashi to prevent snakes' harm; (K) connected with kujur (a religious person in the Nuba Mountains) and spirituality; (L) purposely kill the people.

Sixty respondents (31%) from 14 states responded that they had anecdotes and/or beliefs relating to snakes. The qualitative thematic analysis revealed 12 themes as summarized in Table 4 and Figure 6.

**TABLE 4:** Thematic analysis of stories and beliefs: Frequency and percentage of themes reported in respondents' perceptions of snakes in Sudan.

Theme	Frequency (*n*)	Percentage (%)
Association with *Jinn*	34	47
Avengers of kin	18	25
Guardians of treasures and people	4	6
Connected with birds' behavior	3	4
Bad omen	3	4
Possess a precious jewel in their heads	3	4
Daily connections of bites and venom (Wednesday or Friday)	2	3
Ancestors that should be protected	1	1
Spit their venom on uncovered milk to purposely kill humans	1	1
Are connected with *Kujur* ^a^ and spirituality	1	1
Call on the holly man Sheikh Alkabashi to prevent snakes harm	1	1
Good omens of rain and prosperity	1	1

^a^ *Kujur* = the religious person in the Nuba Mountains. It is formed of two words: Kuj = “to hung” and oor = “spirit”.

Snakes were most commonly associated with *Jinn*, spirits inhabiting the earth and capable of assuming various forms and extraordinary powers (Britannica 2025), in 11 states (Frequency = 34; 47%). Another frequent belief was that snakes seek revenge if any of their family members are killed (Frequency = 18; 25%), while other themes, including snakes as ancestors, guardians, killers, or omens, were less common.

Regional variation was pronounced (Figure 6). River Nile state exhibited the greatest thematic diversity, followed by Sennar state, whereas Al Jazirah and West Kordofan reported more restricted, localized beliefs. These patterns indicate that while some narratives, such as *Jinn* associations, are widely shared, others are region‐specific, shaped by local cultural and social contexts.

### 3.3 Traditional Treatment

Knowledge and perception of traditional treatments varied. The most common responses included making an incision at the bite site and sucking out venom (*n* = 60, 31%), followed by those who use different types of treatments (*n* = 55, 29%), those who have no knowledge of traditional treatments (*n* = 37, 19%), and 14% (*n* = 25) reported snake fang extraction; of these, 24 participants used plant poultices for extraction, with the exception of one who reported the use of frogs. A further 5% of respondents indicated the use of the venom stone, which is believed to be inherited within families and used to extract venom from the bite site. The black stone is placed directly over the bite site, where it reportedly adheres until it has “sucked out” the venom and then detaches naturally. Only 42 respondents reported that they knew the traditional treatment source; 88% (*n* = 37) of them reported a local source, whereas imported traditional treatment was reported by 12% (*n* = 5). Various herbal remedies were identified, including *Citrullus colocynthis* (10%), *Calotropis procera* (6%), *Azadirachta indica* (3%), *Trigonella foenum‐graecum* (3%), and *Ziziphus spina christi* (3%).

### 3.4 Notable Beliefs and Extracted Themes

Examples of notable beliefs that were common and widely held across the study population include:

1. “The snake records the image of its killer in its eyes, and its relatives will seek revenge.”
2. “Large snakes are believed to be a type of *jinn*.”
3. “There are Muslim and non‐Muslim snakes; say ‘*Allahu Akbar’* (God is Great) to distinguish them.”
4. “Snakes follow the righteous and guard gold.”
5. “My grandmother used to say that a snake would come and sit under the water clay pot (*Zeir*). She would prevent anyone from reaching it or disturbing it, because it was her late grandfather, the Faki (traditional Muslim saint), who had passed away. The snake would come in his form, greeting the family, reassuring them, and protecting them as well. They believed that the descendants of a Muslim saint's (Faki) family, after death, would return in the form of animals that carry a sense of awe or fear, such as the snake.”
6. “Some snakes bite only on Fridays or Wednesdays; some can break nasal passages.”

For a comprehensive overview of the most frequently reported beliefs, see Table 4.

### 3.5 Additional Insights

Where participants were invited to share additional thoughts, their responses clustered into seven main themes. First, many noted limited knowledge about snakes, especially regarding species identification, appropriate first‐aid measures, and preventive practices. This lack of information led to a strong call for school‐based lessons, rural awareness activities, and wider public campaigns, with an emphasis on combining traditional and scientific knowledge.

Respondents expressed varied views on the ecological roles of snakes. Some recognized their contribution to controlling rodents and insects and supporting overall ecological balance, while others stressed that snakes should be conserved as part of Sudan's natural heritage.

Strong attention was given to antivenoms and medical care. Participants advocated for improving local production and availability of antivenoms and raised concerns about harmful first‐aid practices—such as cutting the wound, attempting to extract venom, or using phone subscriber identity module (SIM) cards under the belief that copper can remove venom. They also highlighted the need for healthcare workers to be trained to distinguish between venomous and nonvenomous species and deliver appropriate treatment.

Safety, prevention, and first aid were also at the center of respondents' attention. Participants emphasized the importance of targeted awareness efforts on snakebite prevention and calm reactions during incidents, particularly for farmers during planting seasons and in other high‐risk settings. Encounters were often associated with agricultural work, construction activities, and environmental changes.

Cultural beliefs and myths were strongly present in this section also. Respondents mentioned associations between snakes and jinn, hidden treasures, and witchcraft, as well as beliefs about how venom acts or can be used. Attitudes toward snakes ranged from fear to respect and curiosity, reflecting the complex cultural context surrounding them.

Economic and utilitarian uses were also present. Some participants pointed to potential benefits from using snake meat, skins, or venom for food, leather, and medicinal purposes and expressed interest in approaches that allow sustainable use of these resources.

Finally, participants underscored the need for more scientific research and documentation. They called for studies that accurately identify species using both scientific and local names and recommended further field surveys, particularly in areas affected by conflict or abandonment, before communities return.

## 4 Discussion

Despite the ongoing war in Sudan, which has severely disrupted internet access and telecommunications, we were able to engage 192 participants from 16 states, providing a relatively broad geographic representation. The absence of participants from two Darfur states underscores how conflict‐related security and connectivity barriers can directly limit scientific inquiry and biodiversity studies. Nevertheless, the achieved sample offers meaningful insights into Sudanese perceptions and traditional knowledge about snakes.

The seasonal spike in snake encounters during the rainy season aligns with ecological realities, such as increased prey availability (Luiselli 2006), heightened agricultural activity, and habitat disruptions from flooding, highlighting the need for seasonally timed awareness and prevention campaigns. This is consistent with the findings of Barhadiya et al. (2024), who reported that the incidence of snake encounters is highly seasonal and appears to be primarily associated with monthly variations in rainfall, temperature, and humidity.

Species recognition remains a critical gap: while *Naja* and *Echis* species were often mentioned (Figure 3), many respondents could only identify snakes by color, a behavior that can lead to dangerous misidentification, affecting both snakebite risk and conservation. These findings highlight the urgent need for community‐level educational interventions that support accurate snake identification and safer first‐aid guidelines.

Attitudes toward snake conservation were deeply polarized: approximately one‐third of respondents strongly supported protection, while a similar proportion strongly opposed it. Notably, 65% reported killing snakes indiscriminately. This finding is in accordance with our observation in our Facebook outreach group (TORC 2023–2025) where nearly 90% of snakes submitted for identification had already been killed. This points to deeply divided attitudes toward snake conservation in our sample and suggests that conservation initiatives in Sudan would need targeted awareness programs to bridge the gap, emphasizing snakes' ecological role while addressing fears and cultural concerns. It also reflects global patterns: innate fear coupled with culturally rooted myths and phobias can drive widespread persecution of snakes (Musah et al. 2021).

Cultural narratives, such as association with *jinn*, revenge, or treasure guardianship, were widespread and regionally varied. Such beliefs can either hinder or facilitate conservation, depending on how snakes are culturally framed. Rather than ignoring these narratives, integrating them into culturally resonant public engagement strategies may foster greater local acceptance (Wood et al. 2022).

The reliance on traditional remedies, such as incision, suction, black stone use, or improvised methods like SIM cards, reflects the serious lack of access to effective medical care and antivenom. Despite the World Health Organization's clear guidance discouraging harmful practices such as incision and suction, and evidence from Chippaux and colleagues—demonstrating that the black stone is ineffective in treating envenomation (Chippaux et al. 2007), with recommendations instead emphasizing immobilization, reassurance, and prompt medical transfer, the situation on the ground remains markedly different. Many people continue to rely on practices that are either harmful or of no therapeutic value.

In Cameroon, qualitative research reported that 60%–90% of snakebite victims rely on traditional treatments, such as bite site incision and black‐stone application, due to antivenom unavailability‐ with only 5% of antivenom needs being met by health facilities in some districts (Chuat et al. 2021). Similarly, snakebite victims frequently turn to traditional healers because of cost, distance, and weak healthcare infrastructure, while antivenom supplies remain scarce and unreliable across sub‐Saharan Africa (Berg et al. 2024).

Community calls for local antivenom production and enhanced medical training thus reflect not merely public health aspirations, but foundational requirements for effective conservation messaging. Local production and reliable distribution of antivenom, a key WHO strategic priority (Chippaux et al. 2019), should therefore be prioritized. Strengthening healthcare provider training in snakebite recognition, management, and treatment remains equally essential.

Regional differences were striking: northern regions favored jinn‐related beliefs and revenge myths; western areas emphasized treasure‐related narratives and reported declining snake populations; central regions noted frequent encounters near homes and varied treatments; eastern areas reported more stable snake populations and higher uncertainty (“I don't know” responses). This reinforces the need for localized, culturally tailored awareness strategies rather than blanket campaigns. Community‐based interventions, aligned with WHO's community empowerment strategies (Chippaux et al. 2019), are thus vital for sustainable, locally owned solutions.

Importantly, 95% of respondents supported merging traditional and scientific knowledge, offering a powerful entry point for co‐developed, culturally grounded conservation education. Participants also expressed interest in scientific documentation of species, especially in conflict‐affected areas, signaling both awareness of a biodiversity gap and urgency for baseline monitoring before large‐scale resettlement. This aligns with research in rural Kenya showing that local perceptions of snakes and snakebites—such as the use of black stone or removal of snake “teeth”—need to be directly addressed through culturally adapted educational tools that integrate Local Ecological Knowledge and art forms to shift misconceptions (Wood et al. 2022).

In Sudan, culturally rooted music and artistic approaches have demonstrated substantial influence. A previous environmental awareness initiative employed music to encourage children to protect vegetation and refrain from cutting tree foliage; the resulting song became widely remembered among Sudanese youth and received widespread acclaim, featuring regularly on national television and radio. Creative media—including music, painting, and drama—thus represent effective channels for embedding updated conservation messages and for strengthening community engagement in snakebite prevention and broader ecological stewardship.

Several limitations must be acknowledged here. Self‐reported data may be influenced by recall bias or social desirability. Uneven participation, particularly the absence of responses from two Darfur states, limits representativeness. Reliance on online distribution likely excluded participants with limited internet access or digital literacy, including rural, older, or displaced populations. Recruitment via social media may have introduced selection bias, with respondents not fully representative of the wider population. Additionally, the ongoing conflict in Sudan likely contributed to lower participation in western regions, particularly Darfur states.

## 5 Conclusions and Recommendations

This study highlights the complex interplay between ecological realities, cultural beliefs, and public health responses to snakes in Sudanese communities. Despite logistical challenges, the participation of 192 respondents across 16 states (out of 18) provided valuable insights into perceptions, traditions, and the intersections of conservation and public health. The findings underscore the importance of integrating Local Ecological Knowledge (LEK) with scientific strategies to develop culturally sensitive and effective interventions.

Three main recommendations are suggested here:

The first recommendation emphasizes the need for public health initiatives that integrate evidence‐based first‐aid training into everyday community practices while acknowledging traditional methods in a respectful manner. Such an approach can help ensure cultural relevance and community acceptance. At the same time, enhancing healthcare providers' skills in recognizing, managing, and treating snakebites remains essential for improving patient outcomes and reducing both morbidity and mortality.

The second recommendation focuses on addressing misconceptions about snakes through awareness efforts that recognize their cultural significance. Blending Local Ecological Knowledge with scientific information can support coexistence, deepen public understanding, and encourage appreciation of biodiversity. This dual approach helps correct misinformation without dismissing culturally rooted perspectives.

The third recommendation highlights the importance of culturally tailored education and continued research. Awareness programs should prioritize snake identification, seasonal patterns of encounter, and practical preventive behaviors. Because beliefs and practices vary across regions, context‐specific educational strategies are crucial. Further research is needed to document species knowledge, cultural beliefs, and health behaviors across Sudan's states, providing a foundation for designing future evidence‐based interventions.

Overall, this study provides a foundation for culturally informed, evidence‐based interventions that integrate traditional knowledge with scientific approaches. By addressing public health, conservation, and educational needs in a context‐specific manner, these recommendations aim to improve human‐snake coexistence, reduce snakebite risks, and support sustainable biodiversity management across Sudan.

## Author Contributions

**Rania M. H. Baleela:** conceptualization (equal), data curation (lead), formal analysis (lead), visualization (lead), writing – original draft (lead), writing – review and editing (equal). **Muhammad E. M. O. Elamin:** conceptualization (equal), writing – review and editing (equal). **Abubakr Mohammad:** conceptualization (equal), writing – review and editing (equal). **Sara A. K. Saeed:** conceptualization (equal), writing – review and editing (equal).

## Conflicts of Interest

The authors declare no conflicts of interest.

## Supporting information


**Appendix S1:** ece372959‐sup‐0001‐AppendixS1.pdf.

**Appendix S2:** ece372959‐sup‐0002‐AppendixS2.xlsx.

## Data Availability

The data supporting the findings of this study are available within the article (data sources, tables and figures) and in the Appendix S2.
