# Supplementary material for: Local Ecological Knowledge and Cultural Perceptions of Snakes in Sudan
Source: Ecol Evol. 2026 Jan 18;16(1):e72959. doi: 10.1002/ece3.72959 (PMC12812858; doi:10.1002/ece3.72959)
Supplement: Supplementary file 1 — Appendix S1: ece372959‐sup‐0001‐AppendixS1.pdf. [file ECE3-16-e72959-s001.pdf]

A questionnaire on traditional knowledge about snakes in Sudan

Peace be upon you, and God's mercy and blessings.

Our research team is conducting a study aimed at documenting traditional knowledge about snakes in Sudan. For this purpose, this questionnaire has been prepared. We kindly ask you to read it carefully and then select or provide the required information and respond to the questions.

Your participation is voluntary, and all information you provide will be used solely for scientific research. No one other than the research team will have access to it. Therefore, we kindly ask you to answer accurately and honestly.

All team members are Sudanese researchers. The team consists of two professors from the Faculty of Science at the University of Khartoum, a physician specializing in clinical toxicology, and a researcher specializing in natural diversity.

Completing the questionnaire will take no more than six minutes.

The Research Team

Section 1 Demographics

1. **What is your age group (years)?**
   a) <18
   b) 18-30
   c) 31-50
   d) >50
2. **Gender:**
   a) Female
   b) Male

Sections 2-20:
These sections were dedicated to specifying the state and locality for which the participant would provide information on traditional local knowledge about snakes. Lists of all 18 states of Sudan and their detailed localities was provided. Once the participant selected a state, the questionnaire automatically directed them to the corresponding localities. They then chose the appropriate locality from a drop-down menu before proceeding to the subsequent thematic sections.

Sections 21: knowledge of local ecology and snake diversity

1. Are snakes common in your area?
   ☐ Yes ☐ no
2. Which places do snakes prefer in your area? Select all that apply.

☐ Agricultural lands
☐ Forests
☐ Wetlands
☐ Near houses
☐ Sand dunes

☐ Open areas / wilderness

☐ Other

If you selected "Other" above, please specify:

…………………………………………………………………………………………….

3. In which season do snakes appear more frequently? Select all that apply.

☐ Rainy season

☐ Summer season

☐ Winter

☐ Dry season

☐ All year

4. Do you know any dangerous species of snakes in your area?

Yes ☐ no ☐

If your answer is yes, list the names of the snakes found in your area

…………………………………………………………………………………………….

<u>Section 22: Local knowledge and practices</u>

1. Are there traditional ways to identify snakes in your community?

Yes ☐ no ☐

2. Are there any cultural beliefs or stories associated with snakes? Please share any significant ones.

…………………………………………………………………………………………….

3. How do people in your community usually deal with snakes? Select all that apply

Avoidance ☐ Hunting ☐ Worship ☐ Coexistence ☐ Kill it ☐

4. Are there specific individuals in your community regarded as experts on snakes?

Yes ☐ no ☐ I don't know ☐

5. How do people distinguish between venomous and non-venomous snakes? Select all that apply

☐ According to the colour or patterns

☐ According to the size or shape of the head

☐ They cannot distinguish

☐ I don't know

<u>Section 23: snake behaviour and population dynamics</u>

1. Have you noticed a change in the number of snakes over time?

Stable ☐ Increasing ☐ Decreasing ☐ I don't know ☐

2. Have you noticed any change in the activity or behavior of snakes during the current war?

   Yes ☐ no ☐ I don't know ☐

3. What do you believe snakes feed on?

☐ Rodents

☐ Other snakes

☐ Birds

☐ Insects

☐ Frogs and toads

☐ Other

☐ I don't know

If you selected "Other" above, please specify: ………………………………………………

<u>Sections 24-26: Human - snake interaction and traditional treatment</u>

1. What are the most common reasons for human-snake encounters in your area?

☐ Farming and land preparation

☐ Harvest season

☐ Snakes are found near houses

☐ The presence of birds in the house

☐ While hunting

☐ Other

☐ I don't know

If you selected "Other" above, please specify: ………………………………………………

2. Are snakebites common in your area?

   Yes ☐ no ☐

3. Are there traditional remedies for snakebites in your community?

   Yes ☐ no ☐

4. If you answered "Yes" to the previous question, please choose the traditional treatments used:

☐ Incising the area and sucking out the venom

☐ Using poultice herbs to extract the fangs

☐ Eating certain foods or fasting

☐ Drinking certain drinks

☐ More than one type of treatment is used together

☐ Use venom stone

☐ I don’t know

☐ Other

4.1 The plant used is:

☐ Usher (*Calotropis*)
☐ Colocynth
☐ Neem
☐ *Aristolochia*
☐ Acacia
☐ Fenugreek
☐ Sidr
☐ Aloe
☐ Herbs I don't know the names of

4.2 The drink used is:

☐ Black tea
☐ *Aristolochia* tea
☐ Coffee
☐ Salt in water
☐ Lime
☐ Ghee
☐ Gum Arabic drink
☐ Raw eggs
☐ Mud drink

4.3 Eat/Chew/Fast:

☐ Honey
☐ Toombac (Sudanese snuff)
☐ Fast

4.4 If you selected “Other” above, please specify: ………………………………………………

4.5 If you selected “More than one type of treatment is used together” above, please specify:
………………………………………………

5. Do you know where the practitioner/expert/healer obtains the traditional treatment from?

Yes ☐ no ☐

If you choose "Yes" to the previous question, please specify:

…………………………………………………………………………….

6. The traditional treatment source is:

   Local ☐ Imported ☐ I don't know ☐

<u>Section 27: Awareness and conservation practices</u>

1. Have you or your community received any awareness about snakes or how to handle their bites?

   Yes ☐ no ☐

2. Are there any beliefs or traditional practices that help preserve snake populations?

   Yes ☐ no ☐

If you selected "Yes" above, please specify: ……………………………………………….

3. How does your community feel about the conservation of snakes?

Supportive ☐ Neutral ☐ Opposed to ☐

4. Are there modern scientific or governmental programs for snake research or management in your area?

   Yes ☐ no ☐ I don't know ☐

5. Do you think integrating traditional knowledge with science could improve the protection of both humans and snakes?

   Yes ☐ no ☐

6. From 1-5, where 1 = "I strongly disagree" and 5 = "I strongly agree":What is your opinion on the following statement:

"Snakes are important creatures that should be protected"?

1. I strongly disagree
2. I disagree
3. Neutral
4. I agree
5. I strongly agree

Section 28 Additional insights and consent

Are you willing to participate in future studies about snakes?

Yes ☐ No ☐ Maybe ☐

Do you think this questionnaire covered all the topics related to traditional knowledge about snakes?

Yes ☐ No ☐

Is there anything else you would like to share about snakes or the traditional knowledge related to them?

………………………………………………………………………………………

Consent:

I acknowledge that all the information I have provided in this questionnaire is accurate, and that I have participated in this research voluntarily. I also acknowledge my consent for the researcher to analyze all the information I have provided as part of the collected data for scientific research purposes.

You may choose the option that best protects your privacy should we publish the results.

☐ I agree to have my name mentioned in any published research or conference as a reference.

☐ I do not agree to have my name mentioned in any published research or conference as a reference.

We thank you sincerely for answering the questions and reaching the end of the questionnaire. We will make every effort to analyse the data and present and share the results as soon as possible.

If you have any comments or additional notes, please kindly provide them here…………
